# Inverse Piezoresistive Nanocomposite Sensors for Identifying Human Sitting Posture

**DOI:** 10.3390/s18061745

**Published:** 2018-05-29

**Authors:** Zhe Qian, Anton E. Bowden, Dong Zhang, Jia Wan, Wei Liu, Xiao Li, Daniel Baradoy, David T. Fullwood

**Affiliations:** 1 School of Electronics and Information Technology, Sun Yat-sen University, Guangzhou 510006, China; qianzh5@mail2.sysu.edu.cn (Z.Q.); zhangd@mail.sysu.edu.cn (D.Z.); wanj8@mail2.sysu.edu.cn (J.W.); liuw75@mail2.sysu.edu.cn (W.L.); lixiao20172018@gmail.com (X.L.); 2 SYSU-CMU Shunde International Joint Research Institute, Shunde, Foshan 528399, China; 3 Department of Mechanical Engineering, Brigham Young University, Provo, UT 84602, USA; danbaradoy@hotmail.com (D.B.); dfullwood@byu.edu (D.T.F.)

**Keywords:** sitting posture, inverse piezoresistive nanocomposite sensor, strain gauge, BP neural network, posture identification

## Abstract

Sitting posture is the position in which one holds his/her body upright against gravity while sitting. Poor sitting posture is regarded as an aggravating factor for various diseases. In this paper, we present an inverse piezoresistive nanocomposite sensor, and related deciphering neural network, as a new tool to identify human sitting postures accurately. As a low power consumption device, the proposed tool has simple structure, and is easy to use. The strain gauge is attached to the back of the user to acquire sitting data. A three-layer BP neural network is employed to distinguish normal sitting posture, slight hunchback and severe hunchback according to the acquired data. Experimental results show that our method is both realizable and effective, achieving 98.75% posture identification accuracy. This successful application of inverse piezoresistive nanocomposite sensors reveals that the method could potentially be used for monitoring of diverse physiological parameters in the future.

## 1. Introduction

Sitting may occupy up to half of an adult’s workday in developed countries and poor sitting posture has been linked with increased human health risks [1]. Research shows chronic poor sitting posture has been correlated with spinal pain, poor spinal health and the risk of future spinal problems [2,3], as the unevenly increased pressure distribution may induce lumbar degeneration [4]. Poor reading posture has been connected with development of myopia [5]. Work-related musculoskeletal disorders [6,7,8] are widespread among office workers, especially lower back pain [9,10,11]. Many studies have shown that there is a close connection between poor sitting posture and the formation and development of musculoskeletal disorders [12,13,14]. The negative effects on health caused by poor sitting posture not only affect patients’ physical health and working abilities, but also bring serious financial burdens related to medical care [15]. As more and more work in modern society has transitioned to an office environment, identifying and improving human sitting posture becomes particularly critical.

Traditional research on sitting posture has been primarily evaluated using computer vision techniques. Jaimes and Liu [16] proposed a system to monitor a computer user’s posture and activities in front of the computer by using a camera and a microphone which were placed in front of a computer work area. However, this method is sensitive to light contrast and its use is largely restricted to office environments. Paliyawan et al. [17] focused on the detection of sitting posture for office workers by performing data mining classification on the real-time skeleton data stream captured by a single Kinect camera. This complex and relatively expensive system could effectively monitor the user’s posture with an accuracy of 98%. Recently, parallel efforts have emerged to directly measure sitting posture using a variety of sensor technologies. Aissaoui et al. [18] developed a pressure distribution test system which used a large number of pressure sensors to accurately measure the pressure distribution map of the seat. Ma et al. [19] presented a cushion-based pressure sensors posture recognition system with a posture recognition accuracy of over 99%. Roh et al. [20] proposed a lower-cost version using 4 low-cost load cells that achieved over 97% classification accuracy. Although the sitting posture recognition accuracy of these systems is very high, costs have also been high and portability of these options (i.e., a truly mobile posture recognition system that travels with the user throughout the day) has not yet been achieved, although the textile pressure sensor system very recently demonstrated by Kim et al. [21] holds promise. Thus, this work is motivated by a need for an inexpensive, mobile posture recognition system that can robustly track and inform the user as to their posture status. 

In recent years, nanocomposite sensors [22,23,24] have become a new research hotspot. In particular, nancomposites which leverage conductive materials embedded in flexible substrates have shown promise in applications which measure the daily activities of the human body and convert them into electrical signals which can be digitally analyzed [25,26,27]. One disadvantage of virtually all of these sensors has been a low resting resistance [28]. When the strain is low (i.e., most of the time), the resistance of the sensor is low and the power draw for the strain sensing circuit is high. In 2010, our team at Brigham Young University began working on creating inverse piezoresistive nanocomposite sensors [29,30,31,32] which exhibit several important properties that make them good candidates for wearable devices to measure the sitting posture of human beings. These sensors are comprised of dispersed nickel nanostrands (a three-dimensional, branched nickel structure with a nanoscale diameter) and nickel-coated carbon fibers within a silicone matrix. One of the goals of the present work was to identify the specific sensor composition appropriate for the proposed sitting posture monitoring application.

In particular, the sensors exhibit massive inverse piezoresistivity (sometimes referred to as “negative” piezoresistivity) with a high resting resistance. The electrical resistance drops from a resting resistance of over 200 MΩ to 10 Ω with increased strain (over 7 orders of magnitude in resistance change), thus minimizing power consumption during low-strain conditions in use, and exhibiting a very high signal-to-noise ratio. Gauge factors of over 2500 have been achieved with up to 100% strain to failure. A challenge of using these sensors is that the piezoresistive behavior exhibits both non-monotonic as well as nonlinear characteristics. Thus, interpreting the data from the sensors benefits from a machine-learning approach. In this paper, these sensors were optimized and subsequently incorporated as the sensing component of a new wearable, skin-mountable and stretchable device for accurate identification of human sitting postures. The purpose of this work was to design, implement, and validate the use of this device (mobile posture monitor) for detecting, analyzing, and providing real-time feedback on human sitting posture. 

## 2. Materials and Methods 

### 2.1. System Structure

**Figure 1:**
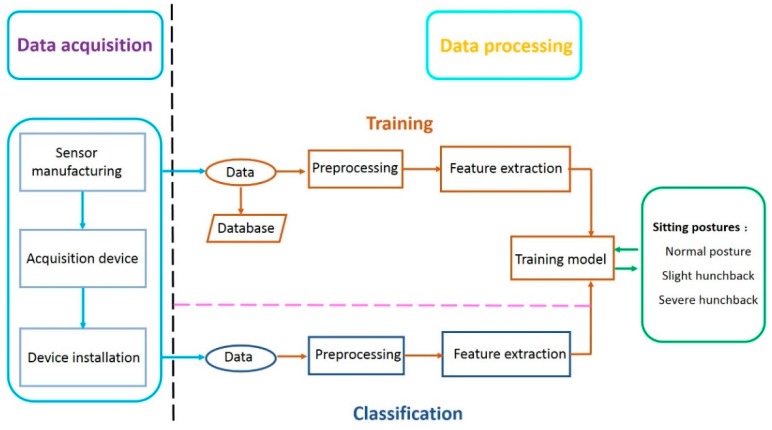
The structure of the proposed system.

The structure of the mobile posture monitor is depicted in Figure 1. The system consists of two major components: the data acquisition unit and the data processing unit. The data acquisition unit is attached on the skin of the lower back of the user and measures the spinal position of the user. The measured signal is then transmitted to the data processing unit. The data processing unit processes the raw data and extracts signal features which characterize distinct patterns of sitting posture. With the extracted features, a model for classification is formed in the training phase of the data processing software. The resultant model is employed in the classification phase to sort the acquired signal features into a predefined class associated with a particular sitting posture. 

### 2.2. Data Acquisition

#### 2.2.1. Sensor Manufacturing

The specific nanocomposite sensor composition used in the mobile posture monitor was comprised of a commercially available silicone matrix material (Ecoflex 00-30, Smooth-On Corporation, Macungie, PA, USA) with nickel nanostrands (Conductive Composites, Inc., Heber City, UT, USA) and nickel-coated carbon fibers (1.0 mm length, 12 k carbon fiber with 20 wt % nickel coating, Conductive Composites, Inc.). The volume fractions of the nickel nanostrands and nickel-coated carbon fibers were optimized using a Design of Experiments (DOE) approach to optimize the electro-mechanical response, in terms of maximizing the gauge factor (sensitivity to strain) and minimizing the critical strain (strain-level beyond which resistance decreases monotonically with further strain). Furthermore, several further filters were used to select an appropriate gauge: 1. the sensor must be readily manufacturable; 2. the mechanical properties must be consistent with the desired application (low stiffness); and 3. the sensor should operate, without failure, within the anticipated strain ranges that would be imposed upon it during different sitting postures (i.e., 0–10% strain nominally). A single-sample, block design experimental approach was used and volume fractions from 3–11% nickel nanostrands and 0.5–3% nickel-coated carbon fibers were evaluated (Table 1).

**Figure 2:**
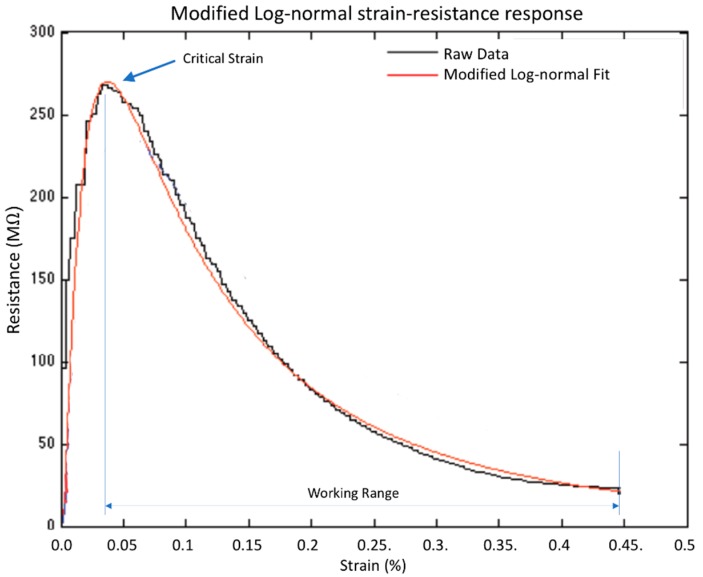
The piezoresistive response of the sensors was found to fit a modified log-normal response, increasing in resistance between 0 to the critical strain, followed by a decrease in resistance over the working strain range of the sensor.

In general, all conductive samples exhibited a modified log-normal, strain-resistance response (Equation (1), Figure 2), and achieved an average *R*^2^ fit value of 0.93.

(1) $$R=d\cdot \left[\frac{c}{a\varepsilon \sqrt{2\pi }}\right]\exp\left(-\frac{{\left(\ln\left(\frac{\varepsilon }{c}\right)\right)}^{2}}{2a^{2}}\right)\;$$

where *R* is the resistance of the sample, ε is the strain, *a* is the log-normal shape parameter, *c* and *d* are scaling parameters. Samples that contained lower than 5% nickel nanostrands or 0.5% nickel-coated carbon fiber were not found to be conductive at reasonable strain values. Most samples with 11% NiNs had both very high initial resistance, as well as very large critical strains, producing samples that failed before achieving conductivity. Samples with greater than 11% nickel nanostrands (and not included in the DOE) were physically poor samples, due to the large amount of filler preventing consistent curing of the silicone. Like many nanopolymeric composites, the sensors exhibit an electromechanical drift that is dramatically reduced when a pre-conditioning protocol is used, thus each sample was pre-conditioned 4 times to 50% strain, and then the electro-mechanical response of the 5th cycle to 50% strain was recorded and assessed for gauge factor and for critical strain. Following this pre-conditioning protocol, the electromechanical drift was virtually eliminated. Mechanical strain was applied using a HANDPI HP-500 tensile tester and electrical resistance was obtained using a Victor VC86E digital multimeter with a maximum resistance capability of 220 mega-ohm. 

Based upon the data from all the conductive samples in the block DOE (i.e., 10 distinct sample compositions with full-range strain-resistance response), parameter values *a*, *b* and *c*, from Equation (1) were identified as functions of NCCF and NINs volume fractions; the *p*-values for these fits were all below 0.05, indicating high confidence in the model. Assessing the gauge factor and critical strain associated with the resultant model, final volume fractions of 11% screened nickel nanostrands and 2% nickel-coated carbon fiber were identified by the optimization criteria as being most favorable and were used as the final sensor composition. 

**Figure 3:**
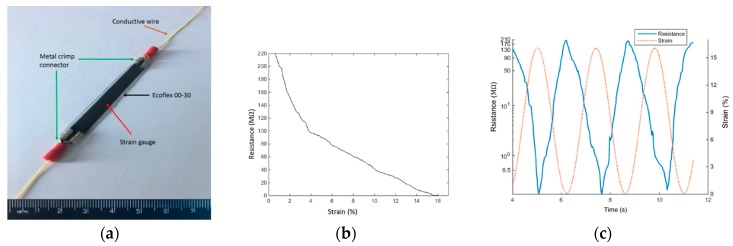
Optimized sensor (**a**) complete sensor package; (**b**) piezoresistive response; (**c**) cyclic piezoresistive response of the sensor.

The optimized sensor was fixed at both ends into metal crimp connectors and connected with conductive wires. Then the complete gauge assembly was covered with a thin layer of Ecoflex 00-30 to seal it from environmental effects (Figure 3a). The complete sensor exhibited the strain/resistance response shown in Figure 3b. The gauge factor of the sensor varied with strain, averaging 25.5 from 0–4% strain and 9.2 from 4–15% strain. The sensor was capable of accurately measuring strains up to 100% strain, but values above 50% strain induced high levels of sensor drift within a short number (50–100) of cycles. Cyclic strains from 0–20% (Figure 3c) were applied for 1000 cycles at 0.4 Hz without any observed permanent changes in the material or piezoresistive response. Relaxation time was not measured, but was assumed to be similar to that of the native cross-linked elastomer (tens of seconds), and anecdotally was not observed to influence the sensor performance in this application. Finally, the completed, sealed nanocomposite gauge assembly was connected in series with a resistor in a simple “voltage-divider” circuit. Testing of the completed device took place over the course of several weeks, during which time no changes in response of the system were noted.

#### 2.2.2. Acquisition Device

**Figure 4:**
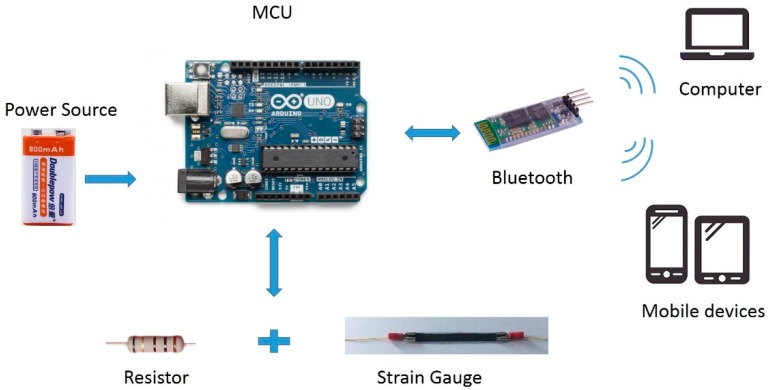
The hardware components of the mobile posture monitor.

As shown in Figure 4, the acquisition device was comprised of a microcontroller (Arduino Uno), a power source (9 V battery), and a wireless Bluetooth communication module. 

**Figure 5:**
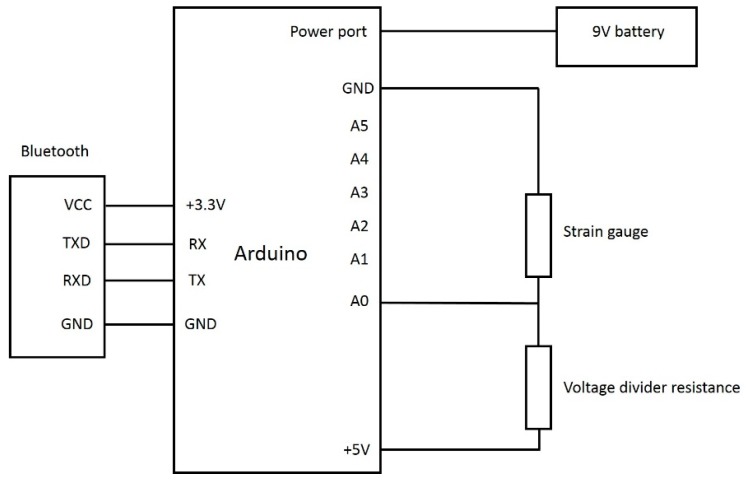
The Circuit diagram of the hardware part.

Figure 5 shows a schematic view of the hardware configuration. Changes in the sitting posture of the user induced deformation of the strain gauge, which reduced its electrical resistance and caused a voltage change. The voltage signal was measured by the microcontroller and scaled between 0 and 1023 using standard A/D conversion techniques. Data was continuously sampled at 50 Hz and the measurement at any given time point was taken as the rolling average of the previous two second interval. The posture data was transmitted to the user’s nearby mobile device through a directly attached Bluetooth HC-06 module.

**Figure 6:**
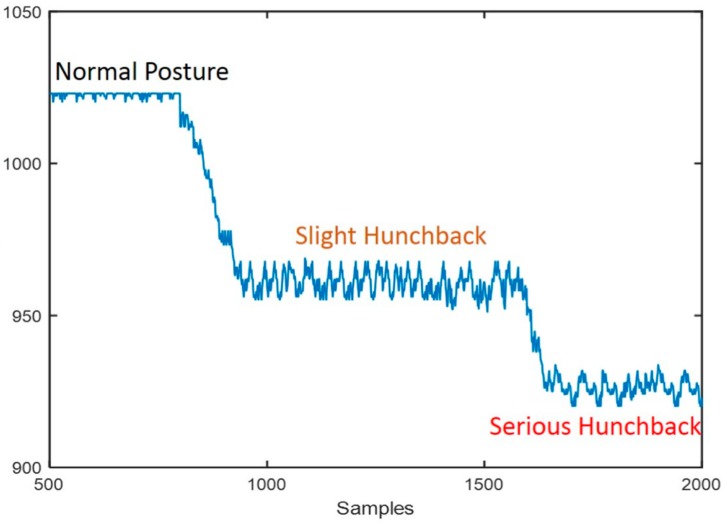
Data output from a test session with a user who changed posture form normal to hunchbacked. The inverse piezoresistivity results in distinct patterns for different sitting postures.

Figure 6 shows the measured data from a typical testing session. It can be seen that changes in back posture led to increased deformation of the strain gauge, and an associated decrease in the resistance of the gauge due to its inverse piezoresistivity.

#### 2.2.3. Device Installation

**Figure 7:**
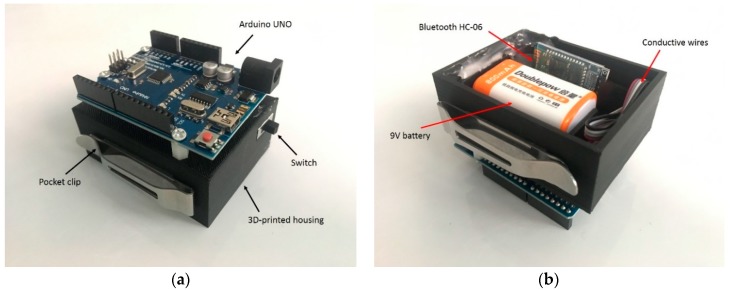
The 3D-printed electronics housing: (**a**) top view of the housing; (**b**) bottom view of the housing.

In order to improve the portability of the system, a 3D printed housing was created to contain the electronic components of the device (the microcontroller, battery and Bluetooth module). The housing was attached to the users’ belts or pockets by means of an integrated pocket clip (see Figure 7), enabling a completely mobile solution that could be used outside of the laboratory environment.

**Figure 8:**
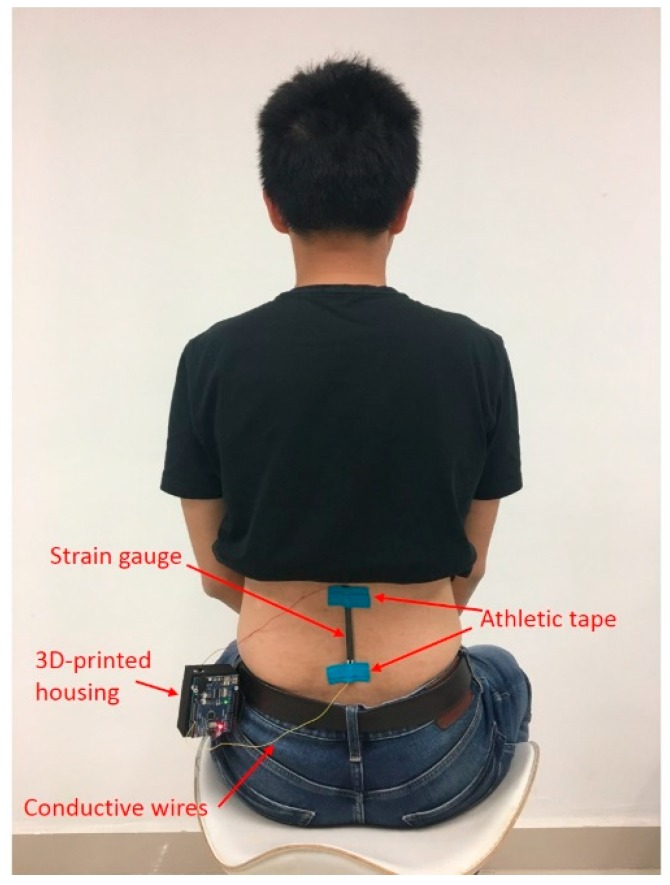
Wearable device for identifying sitting postures.

The silicone material of the sensor naturally adheres to the skin, but was secured by off-the-shelf kinesiology tape, which was used to attach the strain gauge to the lower back of the subject, as shown in Figure 8. The strain gauge was affixed to a region at a midpoint between the two shoulder blades of the subject, and along the lumbar lordotic curve of the spine [33,34,35].

### 2.3. Data Processing

#### 2.3.1. Preprocessing

The noise caused by jitter of the human body, and the measurement noise of the system, interfere with the signal to negatively influence the recognition of sitting posture. In order to decrease the impact of the noise and get more accurate identification results, some preprocessing was performed before extracting features. A given sample point was obtained as the moving average of *N* successive data points. The filtered value is determined by Equation (2):

(2) $$x_{i}^{\prime }=\frac{1}{N}\overset{n=k}{\underset{n=-k}{\sum }}x_{i+n}\;\;\;\left(N=2k+1\right)$$

where $x_{i}$ represents the ith data point, N denotes the number of points in the moving average, and $x_{i}^{\prime }$ represents the filtered value of the ith sampling point.

**Figure 9:**
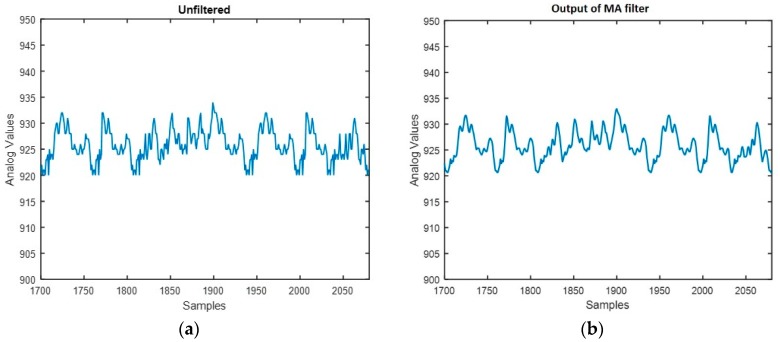
The sampling signal before and after filtering: (**a**) sampling signal before filtering; (**b**) sampling signal after filtering.

Figure 9a,b shows the comparison of the samples before and after filtering. It can be seen that the noise in the sitting signal was removed by the moving average filter. The filtered signal was then passed along to the feature extraction process. 

#### 2.3.2. Feature Extraction

Efficient feature extraction from the measured signals plays a critical role in identifying human sitting posture. The quality of the feature extraction directly affects the identification rate of the classifier. 

**Figure 10:**
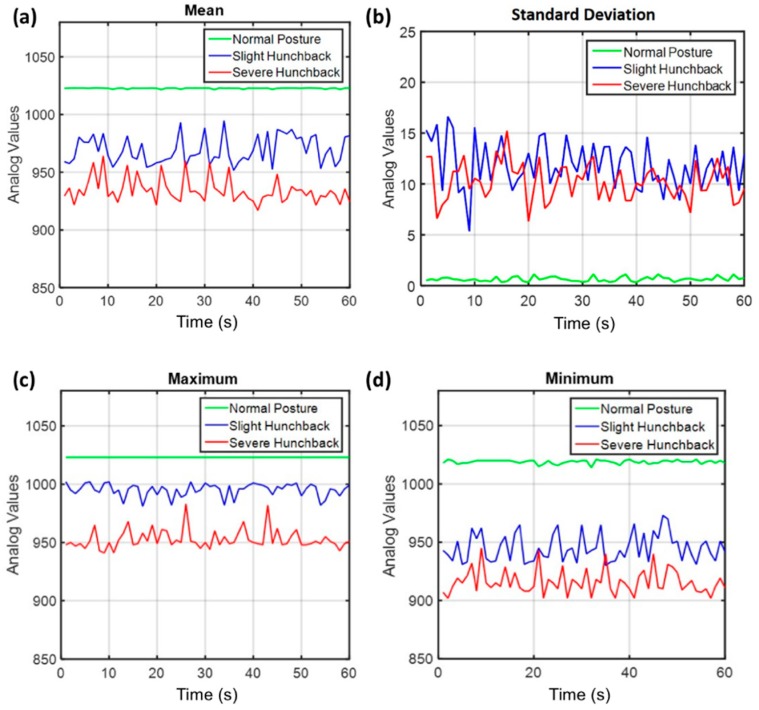
The four time-domain features: (**a**) the mean of different sitting signals; (**b**) the standard deviation of different sitting signals; (**c**) the maximum of different sitting signals; (**d**) the minimum of different sitting signals. Units for times (*x*-axis) are in seconds.

We investigated the mean, standard deviation, maximum, and minimum of the acquired signals and identified three distinct sitting posture patterns, i.e., normal posture, slight hunchback, and severe hunchback. As depicted in Figure 10, normal posture, slight hunchback and severe hunchback show distinct pattern on the mean, the standard deviation, the maximum, and the minimum of the acquired signals. Although each of these features is individually almost able to differentiate the three patterns of sitting posture, there is overlap between the patterns of slight hunchback and severe hunchback when only a single feature is employed, which substantially decreased the identification rate. Thus we combined the mean, standard deviation, maximum, and minimum of the acquired signals into a feature vector, which was passed along to a machine learning classification process.

#### 2.3.3. Classification

The extracted feature vectors were normalized before passing to an Artificial Neural Network (ANN) which was used as a classifier to distinguish patterns of sitting posture (Equation (3)).

(3) $$f_{k}^{\prime }=\left(f_{k}-f_{min}\right)/\left(f_{max}-f_{min}\right)$$

where $f_{min}$, and $f_{max}\;$ represent the elements with minimum and maximum value within the 2-second feature vector respectively. $f_{k}$ represents the kth element, and $f_{k}^{\prime }$ denotes the kth normalized element, k∈{1, 2, 3, 4}.

As an efficient computational model, ANN [36,37] is widely employed in pattern recognition, signal processing, and automatic control. Among the many neural networks, the BP (Back Propagation) neural network plays an important role in many practical applications [38,39] for its simple structure and straightforward implementation. In this paper, we utilized a three-layer BP neural network to construct our classifier. The employed BP neural network consists of an input layer, an output layer and a hidden layer. The performance of a BP neural network depends on the parameters of the architecture, such as the number of neurons in the hidden layer, and the training function. As there was not yet a pre-defined procedure for deciding the parameters of the network structure, the critical parameters were set using an empirical approach, as shown in Table 2.

## 3. Results and Discussion

### 3.1. Data Set

Considering that people of different age, gender, height and weight may produce distinct strain values over the measured back region in the same sitting posture, and thereby send distinct signals to the acquisition device, we recruited 35 volunteers (17 female and 18 male) as subjects for the posture analysis experiment. All subjects gave their informed consent for inclusion before they participated in the study. The study was conducted in accordance with the Declaration of Helsinki, and the protocol was approved by the Institutional Review Board of Brigham Young University (Protocol X15260). Demographics of the volunteers are provided in Table 3.

**Figure 11:**
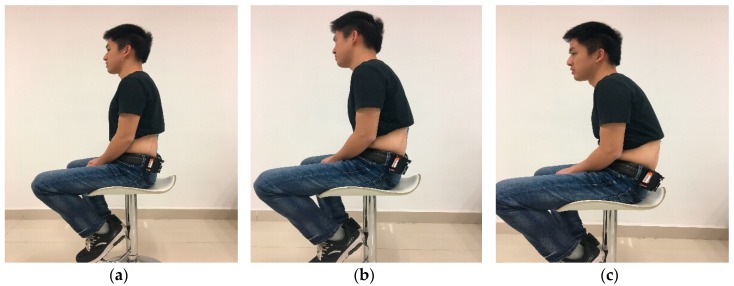
Three kinds of sitting postures: (**a**) normal sitting posture; (**b**) slight hunchback; (**c**) severe hunchback.

The mobile posture monitor was used to measure different sitting postures for each volunteer. As shown in Figure 11, the subject changed from normal sitting posture to slight hunchback and then to severe hunchback during each measurement. In order to eliminate the noise caused by human jitter as much as possible, each sitting posture was maintained for 10 s. Thus, for each sitting posture, there were 500 data points obtained by the acquisition device at 50 Hz. This data was filtered (Equation (2)), and then each window of 100 samples was used to generate a feature vector. In this way, 15 four-dimensional feature vectors were obtained from each experiment. Each experiment was performed with each volunteer subject. Finally, we set up a data set composed of 5250 four-dimensional feature vectors acquired from testers in normal sitting posture, slight hunchback, and severe hunchback. In the classification step, we randomly selected 80% of the data as the training set to train the BP neural network model, and the remaining 20% of the data as the test set to evaluate the performance of proposed system.

### 3.2. Neuron Selection

In the BP neural network, the selection of the number of neurons in the hidden layer has a great influence on the performance of the neural network. In general, the empirical formula for determining the number of neurons in the three-layer BP neural network is given by Equation (4):

(4) $$k=\sqrt{m+n}+a$$

where k represents the number of neurons in hidden layer, n denotes the number of neurons in input layer, m represents the number of neurons in output layer, and a represents a constant between 1 and 10.

**Figure 12:**
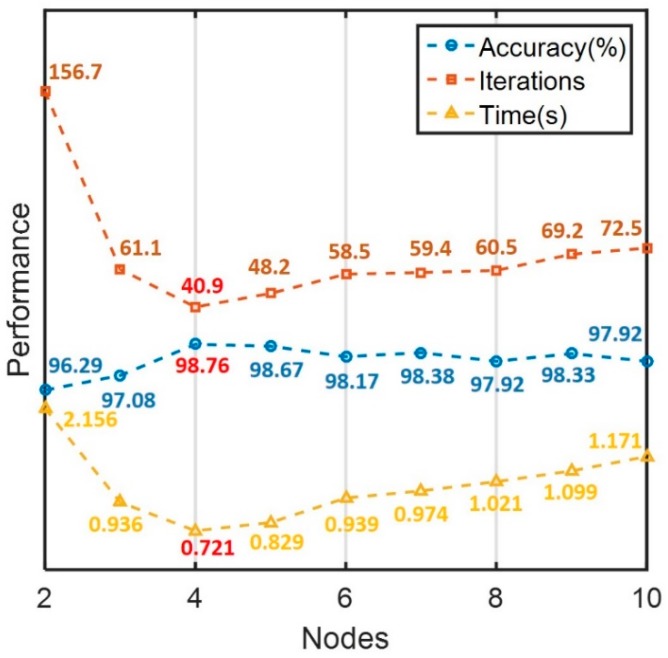
The influence of different number of hidden neurons on the performance of the model.

The above empirical formula is only for reference. However, in the design of a practical network, the optimal number of neurons is usually determined using an empirical construction method. First, we set up a hidden layer with the number of neurons based on Equation (4). If the model failed to provide the required performance, then the number of neurons was gradually increased until an effective and efficient recognition process was achieved. As shown in Figure 12, when too few neurons were employed, the accuracy of the classifier was rather poor despite an extended solution time. When too many neurons were used in the hidden layer, the accuracy of classification was not improved, despite increased iterations and solution time (i.e., overfitting of the model). Based on our empirical process, the performance of the network model was best when the number of neurons in hidden layer was close to the dimension of the feature vectors. 

### 3.3. Training Function Selection

The BP neural network trains the network model through the training functions. Typical training functions (corresponding Matlab functions designated in parentheses) include: gradient descent (traingd), gradient descent with momentum (traingdm), gradient descent with adaptive learning (traingda), resilient backpropagation (trainrp), and Levenberg-Marquardt backpropagation (trainlm). In practical applications, training functions are usually determined based on performance for the specific problems and samples. As shown in Table 4, different training functions impacted the performance of the BP neural model in terms of accuracy, training times, and training error (commonly referred to as mean square error). In our experiment, the model with trainlm showed superior performance to other training functions in all aspects, as it achieved higher accuracy and lower error with fewer iterations.

### 3.4. Performance Evaluation

**Figure 13:**
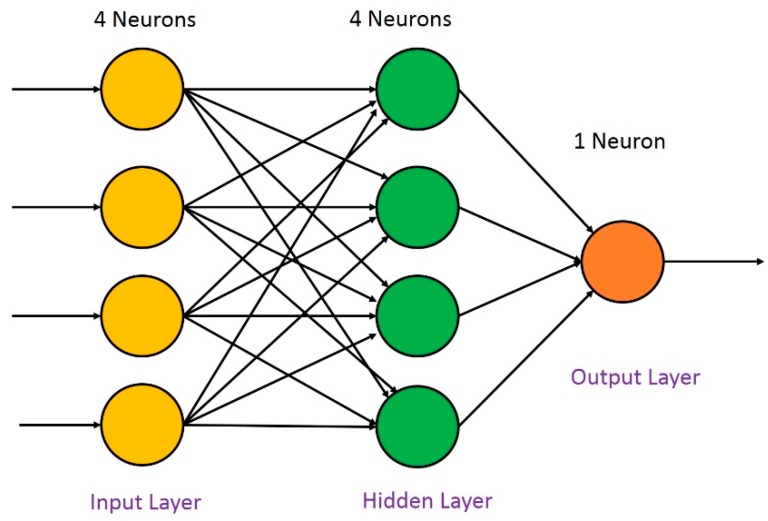
Structure of the BP neural network.

**Figure 14:**
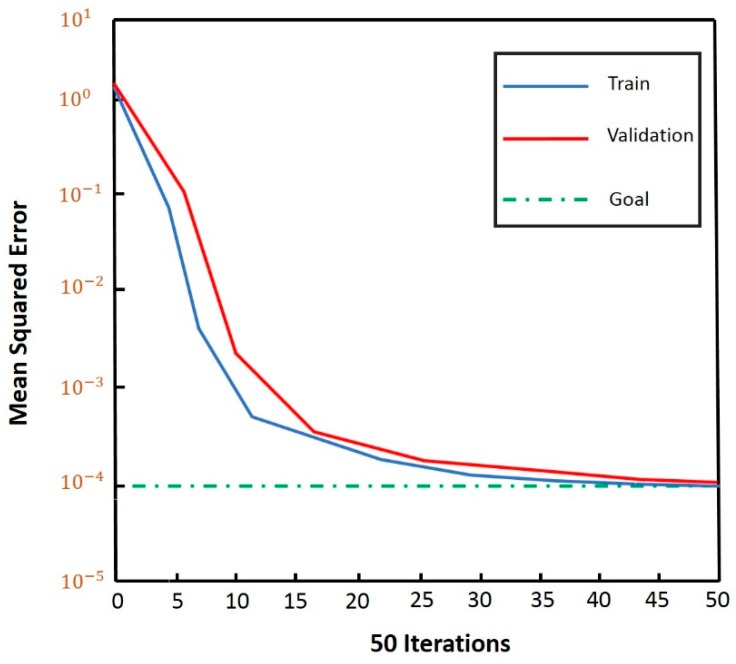
The mean squared error decreases with the iterations of model training.

In this work, a three-layer BP neural network model was adopted (see Figure 13). As discussed, the number of neurons in the input layer was set to equal the feature dimension (i.e., 4 neurons). Three sitting postures (normal sitting posture, slight hunchback and severe hunchback) were classified by the model, based on the experiments described in Section 3.2 and Section 3.3. As shown in Figure 14, the error of the validation set was virtually equivalent to the error of the training set after fifty iterations, which demonstrated that the BP neural network was performing successfully.

A confusion matrix was employed to evaluate the performance of the proposed classifier, as shown in Table 5. In the confusion matrix, the sitting posture of each row represents the true sitting posture, and correspondingly, the sitting posture of each column represents the predicted sitting posture.

The performance shown in the confusion matrix are defined as in Equations (5)–(7):

(5) $$Accuracy=\frac{N_{true}}{N_{total}}$$

(6) $$\;Sensitivity=\frac{T_{P}}{T_{P}+F_{N}}$$

(7) $$Precison=\frac{T_{P}}{T_{P}+F_{P}}$$

where $N_{true}$, and $N_{total}\;$ represent the number of all correctly identified sitting postures and total sitting postures for testing respectively, $T_{P}$ (true positives) denotes the number of correctly identified sitting postures among the true corresponding sitting postures, $F_{N}$ (false negatives) represents the number of incorrectly identified sitting postures among the true corresponding sitting postures, and $F_{P}$ (false positives) denotes the number of falsely identified sitting postures among the postures which are not the true corresponding sitting posture.

Experimental results show the accuracy of the normal sitting posture reached 100%, and the precision and sensitivity for slight hunchback and severe hunchback were above 98%. For the posture of slight hunchback, only seven samples were confused with severe hunchback. While for the posture of severe hunchback, only six samples confused with slight hunchback. An overall accuracy of 98.76% was obtained for the whole system.

## 4. Conclusions

This paper proposed a new tool that utilizes an inverse piezoresistive nanocomposite sensor and neural network to identify human sitting postures (normal posture, slight hunchback and severe hunchback) accurately. The developed low-cost tool has several advantages, including high accuracy, simple design, low power consumption and convenient implementation. We selected the volume fractions of the nickel nanostrands (NiNs) and nickel coated carbon fibers (NCCF) for our strain gauge based on a block DOE, with the specific intent of optimizing and integrating these novel sensors into a sitting posture monitoring application. The gauge was sealed with a silicone rubber coating before the experiments in order to avoid environmental factors such as moisture. The strain gauge was connected in series with a voltage divider resistor to measure the sitting posture data of 35 volunteer subjects. In order to improve the mobility of the system, we designed a small 3D-printed housing that was easily attached on the tester’s clothes. A moving average filter was used to remove the signal noise and was separated into 5250 four-dimensional (mean, standard deviation, maximum and minimum) data sets. Finally, we designed and optimized the three-layer BP neural network with four hidden neurons and Levenberg-Marquardt training function. The experimental data was used to validate the effectiveness of our method, and obtained an overall accuracy of 98.76%. The successful application of inverse piezoresistive nanocomposite sensors to this application indicates that this method holds potential for use in the fields of body parameter monitoring, wearable devices, human-machine interfaces, remote control, virtual reality and so forth.

A limitation of the implementation described in this work is that it has only been evaluated for a single function (sitting posture identification). Extension of the work to provide posture information during sleeping, running, walking, etc. is ongoing. An additional limitation is the current unoptimized device design which includes unneeded bulk and wiring. Future research will also be devoted to further reducing the size of the acquisition device and to communicating with smart terminals for implementation of real-time monitoring and alarm. Section 3.3 describes our process for selecting a training function, however beyond comparisons of training functions, the neural network was not further optimized for learning rate. Future work might benefit from a holistic approach to optimizing both learning rate and training model. 

## Figures and Tables

**Table 1:** Block Design of Experiments. Reported values are Average Gauge Factors (10% Working Strain Range)/Critical Strain. NC = not conductive, MF = material failure prior to reaching critical strain.

	3% NiNs	5% NiNs	6% NiNs	7% NiNs	11% NiNs
0.5% NCCF	NC	NC	NC	NC	NC
0.75% NCCF	NC	NC	8.56/0.23	9.35/0.23	MF
1.0% NCCF	NC	NC	8.61/0.097	8.5/0.20	MF
1.5% NCCF	NC	NC	3.79/0.036	7.63/0.037	MF
2.0% NCCF	NC	5.49/0.12	4.55/0.054	3.08/0.049	13.5/0.006

**Table 2:** Parameters of the BP neural network.

No.	Parameters	Setting
1	Total number of network layers	3 layers
2	Number of hidden layer	1 hidden layer
3	Number of neurons in hidden layer	4 neurons
4	Training function	trainlm
5	Learning rate	0.001

**Table 3:** The composition of subjects.

Gender	Number	Age	Height	Weight
Female	17	20∼32 years old	158 cm∼168 cm	45 kg∼51 kg
Male	18	21∼45 years old	167 cm∼182 cm	55 kg∼92 kg

**Table 4:** The performance of different training functions in the BP neural network model.

Training Functions	Algorithm	Accuracy	Iterations	Mean Square Error
traingd	Gradient Descent	96.63%	14145	0.0268
traingdm	Gradient Descent with Momentum	97.67%	9453	0.0211
traingda	Gradient Descent with Adaptive Learning Rate	97.79%	3040	0.0184
trainrp	Resilient Backpropagation	98.29%	231	0.0094
trainlm	Levenberg-Marquardt	98.76%	43	0.0042

**Table 5:** The confusion matrix of experimental results.

	Normal Posture	Slight Hunchback	Severe Hunchback	Sensitivity
Normal Posture	246	0	0	100%
Slight Hunchback	0	403	7	98.29%
Severe Hunchback	0	6	388	98.48%
Precision	100%	98.53%	98.23%	98.76%
